# What causes the burden of stroke in Scotland? A comparative risk assessment approach linking the Scottish Health Survey to administrative health data

**DOI:** 10.1371/journal.pone.0216350

**Published:** 2019-07-08

**Authors:** Elaine Tod, Gerry McCartney, Colin Fischbacher, Diane Stockton, James Lewsey, Ian Grant, Grant M. A. Wyper, Oscar Mesalles-Naranjo, Mag McFadden, Richard Dobbie

**Affiliations:** 1 NHS Health Scotland, Glasgow, Scotland; 2 Information Services Division (ISD), NHS National Services Scotland, Edinburgh, Scotland; 3 Health Economics & Health Technology Assessment, Institute of Health & Wellbeing, University of Glasgow, Glasgow, Scotland; Harvard Medical School, UNITED STATES

## Abstract

**Background:**

The availability of robust evidence to inform effective public health decision making is becoming increasingly important, particularly in a time of competing health demands and limited resources. Comparative Risk Assessments (CRA) are useful in this regard as they quantify the contribution of modifiable exposures to the disease burden in a population. The aim of this study is to assess the contribution of a range of modifiable exposures to the burden of disease due to stroke, an important public health problem in Scotland.

**Methods:**

We used individual-level response data from eight waves (1995–2012) of the Scottish Health Survey linked to acute hospital discharge records from the Scottish Morbidity Record 01 (SMR01) and cause of death records from the death register. Stroke was defined using the International Classification of Disease (ICD) 9 codes 430–431, 433–4 and 436; and the ICD10 codes I60-61 and I63-64 and stroke incidence was defined as a composite of an individual’s first hospitalisation or death from stroke. A literature review identified exposures causally linked to stroke. Exposures were mapped to the layers of the Dahlgren & Whitehead model of the determinants of health and Population Attributable Fractions were calculated for each exposure deemed a significant causal risk of stroke from a Cox Proportional Hazards Regression model. Population Attributable Fractions were not summed as they may add to more than 100% due to the possibility of a person being exposed to more than one exposure simultaneously.

**Results:**

Overall, the results suggest that socioeconomic factors explain the largest proportion of incident stroke hospitalisations and deaths, after adjustment for confounding. After DAG adjustment, low education explained 38.8% (95% Confidence Interval 26.0% to 49.4%, area deprivation (as measured by the Scottish Index of Multiple Deprivation) 34.9% (95% CI 26.4 to 42.4%), occupational social class differences 30.3% (95% CI 19.4% to 39.8%), high systolic blood pressure 29.6% (95% CI 20.6% to 37.6%), smoking 25.6% (95% CI 17.9% to 32.6%) and area deprivation (as measured by the Carstairs area deprivation Index) 23.5% (95% CI 14.4% to 31.7%), of incident strokes in Scotland after adjustment.

**Conclusion:**

This study provides evidence for prioritising interventions that tackle socioeconomic inequalities as a means of achieving the greatest reduction in avoidable strokes in Scotland. Future work to disentangle the proportion of the effect of deprivation transmitted through intermediate mediators on the pathway between socioeconomic inequalities and stroke may offer additional opportunities to reduce the incidence of stroke in Scotland.

## Introduction

In a period of limited resources and competing health priorities, the availability of robust health evidence to inform effective public health decision making is becoming increasingly important [1]. Burden of Disease studies are useful in this regard as they provide a comprehensive assessment of the health of the population through a single combined measurement of fatal and non-fatal health outcomes. A key element of this is the Comparative Risk Assessment (CRA) which quantifies the contribution of modifiable exposures to the disease burden in a population.

Global Burden of Disease (GBD) studies have been estimating the burden of disease attributable to exposures since 1990, and most recently in 2016 as part of ongoing CRA updates [2–4]. Since the first study, there have been considerable methodological advances facilitating the production of estimates for increasingly smaller geographies with estimates for Scotland being produced in GBD 2015 for the first time [5]. A key limitation of these studies to date has been a degree of reliance on synthetic estimates and, until recently, the restriction of exposures to health behaviours and clinical exposures.

Stroke is an important public health problem in Scotland which can result in death or permanent physical or psychological disability [6]. It is also a substantial cause of social and financial costs for affected individuals, their families and wider society [7]. The Global Burden of Disease (GBD) study 2016 ranked cerebrovascular disease including stroke as the second most common cause of death and disability globally and third most common in Scotland [8]. In comparison, the Scottish Burden of Disease, Injuries and Risk Factors Study ranked stroke as the 7th most common cause of death and disability in Scotland in 2015 [9]. Declines in mortality from stroke have however been observed in most Western European countries [10] and Scotland has also seen an overall decrease in stroke mortality, though it remains high in comparison to other countries of Western Europe [11,12]. The percentage of patients in Scotland surviving for at least 30 days after emergency admission to hospital with a principal diagnosis of stroke increased slightly over the past decade from 80% to 84% across all age groups [13]. Although an increase in survival from stroke is a positive outcome, the substantial morbidity that can result from cerebral impairment means that it remains a leading cause of adult disability in the UK^.^[14]. In addition, stroke incidence is strongly correlated with age, with older adults at highest risk [15]. This means that as the population ages, the burden of disease due to stroke may increase [16].

It is well recognised that stroke is a largely preventable disease and a range of potentially modifiable exposures such as poverty, smoking, poor diet, lack of exercise and alcohol consumption, all of which are prevalent in the Scottish population, have been proposed as causal exposures [17]. Given the high mortality and morbidity stroke causes, there is a need to better understand the exposures which should be the focus of intervention if the burden of disease from stroke is to be reduced. This study quantifies the contribution of a range of modifiable exposures to the burden of disease due to stroke in Scotland using a novel methodological approach linking Scottish Health Survey data to routine administrative health data.

## Methods

### Overview

Population attributable fractions (PAFs) were calculated for a range of exposures using effect size estimates (hazard ratios) systematically adjusted for behavioural and socio-economic exposures deemed potential confounders on the causal pathway between exposure and stroke.

**Fig 1:**
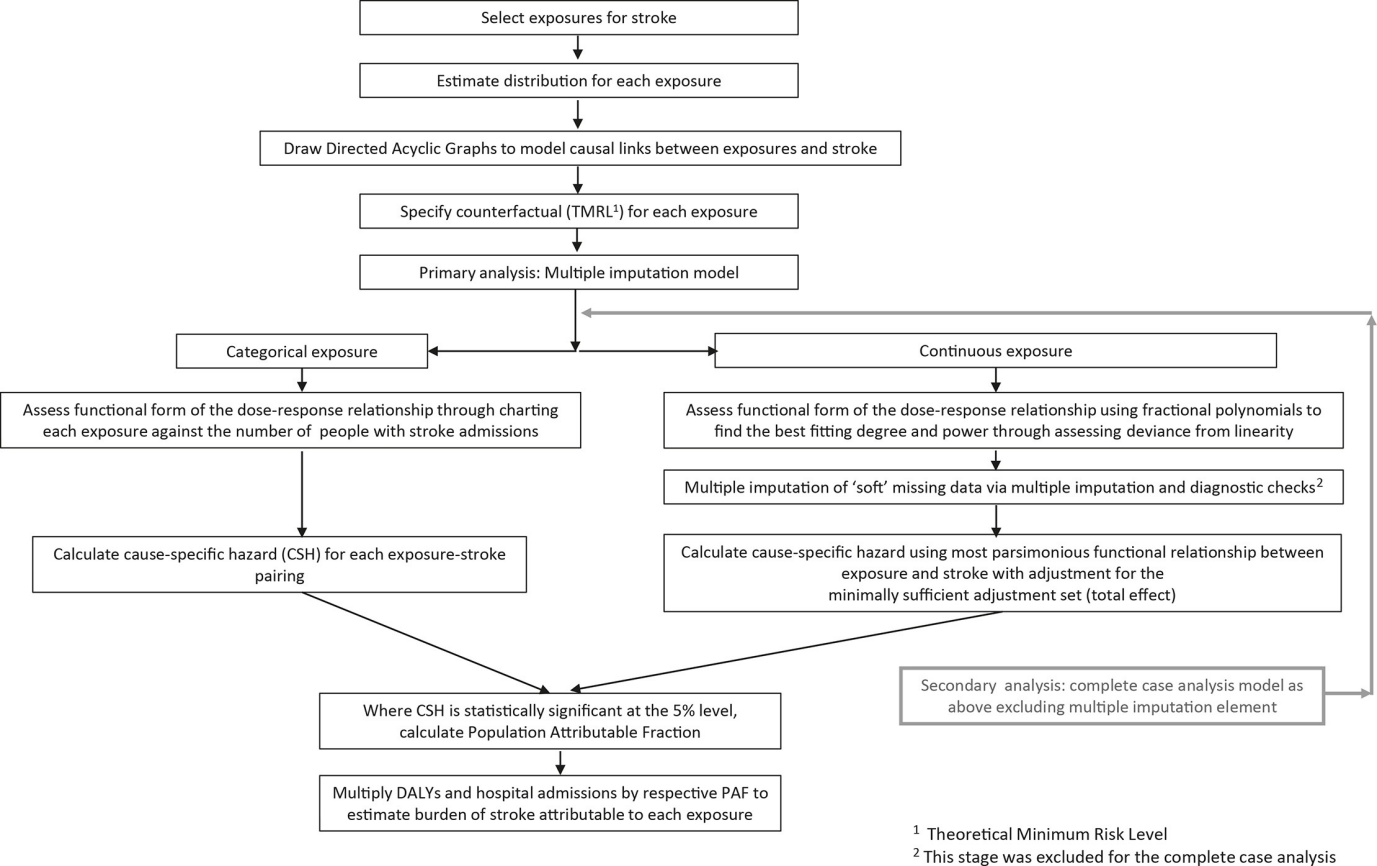
Overview of comparative risk assessment method for estimating the burden of stroke attributable to selected exposures.

We created a series of Directed Acyclic Graphs (DAG) based on the interpretation of the stroke literature by the authors on the causal pathways involved (see S1–S8 Figs). We recognise that our DAGs are limited by the available data and that even the exposures to different aspects of socioeconomic position are themselves caused by other factors across political economy. This was used to develop the adjustments in the modelling such that confounders of all the available exposures on the burden of stroke were identified. The Stata SE statistical package version 13.1 for Windows was used for all analyses [18]. An overview of the comparative risk assessment method used in this study is shown in Fig 1.

### Data sources

Pooled cohort study data was created by linking eight waves of Scottish Health Survey (SHeS) respondents’ data (1995 to 2012) to health service and deaths records. A universal health service identifier, the Community Health Index (CHI), was used to facilitate data linkage. Between 85.6% and 92.8% of SHeS respondents agreed to their survey data being linked to routine hospital admission data across all waves [19]. In total, 51,468 adults who consented to data linkage were able to be followed up in this study. The SHeS sample frame for adults changed from 16–64 years in 1995; to 16–74 years in 1998; and finally to 16+ years from 2003 onwards.

Two data sources on stroke outcomes were linked to the Scottish Health Survey. The first of these was the Scottish Morbidity Record (SMR01) [20], with hospital admissions available for stroke going back to 1981, and the second data source was for death records from National Records of Scotland (NRS).

### Identifying and selecting exposures for stroke

Exposures for stroke were identified through a literature review and mapped to the Dahlgren & Whitehead model of the determinants of health [21]. A list of exposures was compiled from this and reviewed by a panel of stroke experts (see Acknowledgments).

Following identification of exposures for stroke, the potential for measuring each of these using variables from the combined SHeS dataset (1995 to 2012) was assessed. Inclusion of an exposure measure was based on the availability of measures in each survey and comparability across survey waves, the strengths and weaknesses of each measure in estimating exposure and whether there were any changes to survey questions between waves. An exposure was deemed as having no, low, moderate or high potential for modification. See S1 File for the classification criteria.

Some variables changed across the different waves of SHeS. Where this was the case, the salient caveats for interpreting the impact of these differences when combining variables across survey waves are discussed. The variables for highest educational qualification attained could not be combined across survey waves due to significant differences in coding of qualifications over time. To resolve this problem, the qualifications for each wave were mapped to the International Standard Classification for Education (ISCED) 1997; an internationally recognised framework for cross-mapping national qualifications onto a standard, internationally comparable scale [22].

### Defining incident stroke

Stroke was coded using the International Classification of Disease (ICD) 9 codes 430–431, 433–4 and 436; and the ICD10 codes I60-61 and I63-4. This included subarachnoid and intra-cerebral haemorrhage, cerebral artery occlusion and stenosis, cerebral infarction and unspecified strokes; but not Transient Ischaemic Attacks (TIAs). Incidence was defined as death where stroke was recorded as the immediate cause or a hospital admission with a primary diagnosis of stroke. Those who had previously had a stroke, identified through the application of a 10 year look back period to hospital admission records were excluded. In addition, if the participant self-reported previously having had a stroke at any time they were also excluded. There were insufficient events to undertake separate CRAs for stroke sub-types or separately for stroke mortality and morbidity.

### Descriptive analysis

The distribution of exposures in the pooled data-set was explored by age, sex and Scottish Index of Multiple Deprivation (SIMD), a multi-factorial measure of area-based deprivation based on seven domains: income, education, employment, crime, health, housing and geographical access to services [23].

### Missing data

Missing data were defined as either ‘soft’ missing (i.e. those cases which were valid for imputation because individuals did not respond or ‘didn’t know’) or ‘hard’ missing (i.e. those that for whom the question/variable was not applicable). Most missing data were for income, alcohol consumption (almost entirely because they were not asked in the 1995 or 1998 survey waves) and fruit and vegetable consumption. Multiple imputation via chained equations was used to model missing exposure data, starting with 20 imputations [24]. The imputation model for each exposure variable with missing data included all confounders identified from the DAG model and the stroke outcome variable itself. Interaction effects were not included in the imputation model. Validity was checked by plotting the distributions of the observed and imputed values for the continuously measured variables and looking at the proportion of values at each level for categorical variables and then checking whether the imputation algorithm converged (see S1 File).

### Survival analysis

A Cox proportional hazards regression approach was used to examine the relationship between the time to the incident stroke event from the date of the survey interview and exposure to selected modifiable exposures at baseline (survey interview date). As an individual was potentially subject to the competing risk of dying from an event unrelated to stroke, we modelled the cause-specific hazard i.e. the conditional probability that a survey respondent exposed to a particular exposure will be admitted to hospital, or die during the follow-up time-period and that stroke will be the cause [25]. Every exposure was initially modelled adjusting for age and sex. We dropped any variables that did not show significance at p<0.05 between categories to avoid the risk of a type I error.

We avoided spurious causal relationships and confounding by drawing Directed Acyclic Graphs (DAGs) to illustrate causal relationships between each exposure and stroke [26]. We used the DAGitty software [27] with an adjustment set for the *total* effect for a exposure i.e. the blocking of all confounding paths, leaving all direct and mediating causal paths unblocked [27]. Adjustment sets for the direct effect of each exposure mediated through separate pathways was outside the scope of this project (S1 Fig).

The Minimally Sufficient Adjustment Set (MSAS) for the total effect of each exposure on stroke incidence was then used to identify the covariates to adjust for in the final model. This equated to the minimum number of variables that need to be adjusted for in order to control for confounding between the exposure-outcome relationship of interest as identified from the DAG, any subset of which was not sufficient [27]. Calculating the total effect for each exposure facilitated the estimation of the ‘complete’ effect that an exposure may have on stroke risk. The DAG method also allowed for a logical and consistent approach to the degree of adjustment that included socio-economic, behavioural and physiological exposures.

The form of the functional relationship for the continuous exposure-stroke pairings was modelled using fractional polynomials to find the best fitting degree (D) and power (P) through assessing deviance from linearity. All four such variables (BMI, fruit and vegetable consumption, cholesterol and SBP) had a linear function of Degree 1 (D1) and Power 1 (P1). This was deemed the most efficient functional form for the Cox proportional hazards regression.

### Defining the theoretical minimum risk level

The Global Burden of Disease approach for defining a counterfactual level of risk was followed by setting a Theoretical Minimum Risk Exposure Level (TMREL) [2]. This was the level which the evidence suggested posed the minimum health risk to a population [28]. Using the TMREL as the counterfactual indicates the potential for improvement in population health. The TMREL was primarily informed by looking at previous burden of disease/burden of stroke studies [29–32].

As global burden of disease studies have largely focussed primarily on behavioural and clinical exposures, no information was available on setting a TMREL for socio-economic exposures. Instead, the TMREL was informed by reviewing the literature and exploring the exposure-response relationship for the stroke data and socio-economic exposure data in the SHeS-SMR linked dataset. For behavioural and physiological exposures, we chose to use the TMREL identified from Global Burden of Disease studies as these are regularly updated and are based on a systematic review of published and unpublished literature [28].

### Population attributable fractions

The formula used to calculate PAFs was based on the PAF definition of Greenland and Drescher [33] which is recommended for use with survival data [34]. It was calculated using the *punafcc* post-estimation command in Stata. The use of *punafcc* facilitated the inclusion of categorical and continuous exposure variables in the regression model and the calculation of confidence intervals around the PAF. PAFs were not summed as they may add to more than 100% due to the possibility of a person being exposed to more than one exposure simultaneously.

### Burden of stroke attributable to selected exposures

Disability Adjusted Life Years (DALYs), a composite measure of Years of Life Lost and Years Lived with Disability, was calculated by the Scottish Burden of Disease, Injuries and Risk Factors project team [1,35]. A key application of the PAF is the use of this metric in combination with DALYs to estimate the number of DALYs attributable to particular exposures. The total DALYs for stroke were multiplied by the PAF estimates (percentage) to give the number of stroke DALYs, and the reduction in hospital discharge rate, that was attributable to each exposure.

### Data permissions and ethics statement

Ethics approval for SHeS 2012 was given by the National Health Service (NHS) Multi-Centre Research Ethics Committee (reference number 11/WA/0246) and use of linked data was approved by the Privacy Advisory Committee to the Board of NHS National Services Scotland and Registrar General (PAC reference 26/14). This study forms part of the wider National Burden of Diseases, Injuries and Risk Factors Study for Scotland which was initially grant funded by the Scottish Chief Scientist Office (CZH/4/756). Access to the data was granted through the ‘electronic Data Research and Innovation Service’ (eDRIS) of ISD Scotland who ensured that our use of the data and analyses would not breach privacy and confidentiality guidelines. All participants whose data we use from the Scottish Health Survey gave full informed consent to participation in the study and data linkage.

## Results

### Baseline sample characteristics

A total of 49,451 consented survey respondents were followed up over 343,093 person-years (median 4.2 years; range 1 day to 17.8 years). There were more women in the combined sample than men (56.3% and 43.7% respectively); the median age for men and women was the same. There were approximately 20% of respondents in each SIMD quintile with the lowest percentage in the least deprived (17.9%) as outlined in Table 1.

**Table 1:** Demographic characteristics of the 1995–2012 combined SHeS sample.

	Men		Women		Total	
**Population bases**						
Population at risk (N)	21,594	(43.7%)		27,857	(56.3%)	49,451	(100%)	
Person-years at risk						
*Total*	149,583		193,510		343,093	
*Median years (IQR)*	4.2 (2.2 to 13.7)		4.2 (2.3 to 13.8)		4.2 (2.2 to 13.7)	
*Range (years)*	0.1 to 17.7		0.1 to 17.7		0.1 to 17.8	
**Age at survey (years)**						
Median (IQR)	47 (34 to 61)		47 (34 to 61)		47 (34 to 61)	
**SIMD quintile (N)**						
1 (most deprived)	4,162	(41.6%)	5,845	(58.4%)	10,007	(100%)
2	4,308	(43.0%)	5,702	(57.0%)	10,010	(100%)
3	4,498	(44.0%)	5,715	(56.0%)	10,213	(100%)
4	4,624	(44.6%)	5,739	(55.4%)	10,363	(100%)
5 (least deprived)	3,981	(45.1%)	4,837	(54.9%)	8,818	(100%)
**Survey wave (N)**						
1995	3,159	(44.6%)	3,930	(55.4%)	7,089	(100%)
1998	3,527	(44.1%)	4,470	(55.9%)	7,997	(100%)
2003	3,110	(43.9%)	3,979	(56.1%)	7,089	(100%)
2008	2,306	(43.7%)	2,975	(56.3%)	5,281	(100%)
2009	2,642	(43.3%)	3,461	(56.7%)	6,103	(100%)
2010	2,547	(42.6%)	3,433	(57.4%)	5,980	(100%)
2011	2,664	(43.2%)	3,505	(56.8%)	6,169	(100%)
2012	1,639	(43.8%)	2,104	(56.2%)	3,743	(100%)

A total of 575 respondents (1.2%) experienced a stroke hospitalisation as their incident event between the survey interview date and the end of the follow up period. There were 10 additional stroke fatalities (0.02%). Of the other survey respondents, 44,192 (89.4%) were alive and without an incident stroke by the end of the study follow-up period, 3,011 respondents (6.1%) died of a non-stroke related cause and 1,663 (3.4%) emigrated out of Scotland or became otherwise untraceable through CHI.

Of the 585 individuals who experienced a stroke event during the follow up period, 211 were aged 65 years or younger and the remaining 374 were aged 65 years or older. This equates to an age-specific rate of 89 strokes per 100,000 in the under 65 age group and 352 per 100,000 in those aged 65 years or older. These age-specific rates from the SHeS sample were much lower than age-specific hospital discharge rates reported routinely from administrative data for stroke (ICD10 I61, I63 & I64), particularly for adults aged 65 years and over (271.7 per 100,000 in those under 65 years and 3,165.9 per 100,000 in those aged 65 years and over).

### Survival analysis (multiple imputation with chained equations)

Of the 13 exposures included in this study, just over half were still statistically significant predictors (p<0.05) of stroke following estimation of cause-specific hazard ratios (HR) from a Cox-regression model after adjustment for co-variates identified from the DAG model (referred to below as the ‘DAG adjusted model’) (Tables 2 and 3). We also performed a sensitivity analysis without multiple imputation using a complete case analysis. None of the results were substantially altered in any way (not shown).

**Table 2:** Exposure prevalence, stroke event and hazard ratios for selected modifiable socioeconomic exposures, after multiple imputation, adjustment for age, sex and the DAG model.

	Age-sex adjusted model				
Exposure	N Total population*	n No. of incident strokes	HR	95% CI	p-value
**Socio-economic characteristics**					
**Equivalised income**					
1 (Top quintile)	8,793	42	1	(ref)	
2	8,613	65	1.17	0.64 to 2.15	0.601
3	8,270	88	1.41	0.71 to 2.81	0.320
4	8,334	135	1.78	0.98 to 3.20	0.057
5 (lowest quintile)	6,743	102	1.86	0.95 to 3.62	0.069
**SIMD 2012**					
5 (least deprived)	10,012	170	1	(ref)	
4	10,013	129	1.47	1.0 to 2.17	0.053
3	10,216	114	1.83	1.26 to 2.68	0.002
2	10,366	104	1.79	1.23 to 2.60	0.002
1 (most deprived)	8,819	66	2.47	1.74 to 3.51	<0.001
**Carstairs 2001**					
1 (least deprived)	9,295	87	1	(ref)	
2	11,623	120	1.55	1.10 to 2.19	0.012
3	10,427	133	1.41	1.00 to 1.98	0.047
4	9,124	106	1.64	1.15 to 2.36	0.007
5 (most deprived)	8,957	137	2.00	1.44 to 2.79	<0.001
**Social class**					
I. Professional	2,287	16	1	(ref)	
II. Managerial–Technical	13,253	114	1.26	0.63 to 2.55	0.518
IIIN. Skilled–non-manual	10,717	117	1.99	0.98 to 4.05	0.059
IIIM. Skilled–manual	9,050	136	1.85	0.93 to 3.70	0.081
IV. Semi-skilled–manual	8,467	124	2.53	1.25 to 5.12	0.010
Unskilled–manual	3,261	58	2.11	1.01 to 4.46	0.048
**Education**					
Tertiary level (ISCED 5A-5)	10,795	56	1	(ref)	
Post-secondary (ISCED 4-5B)	3,728	20	2.03	1.46 to 2.97	0.057
Upper secondary (ISCED 3A)	7,154	36	1.01	0.59 to 1.79	0.979
Lower secondary (ISCED 3C)	11,648	101	1.41	0.96 to 2.08	0.080
No qualifications (ISCED 0-2A)	14,277	352	2.08	1.46 to 2.97	<0.001
**Employment**					** **
Yes	27,359	154	1	(ref)	
No	20,393	397	1.39	0.97 to 1.98	0.071

* Total population numbers based on the 20^th^ imputation

**Table 3:** Exposure prevalence, stroke event and hazard ratios for selected modifiable individual exposures, after multiple imputation, adjustment for age, sex and the DAG mode.

Individual behaviours								
Exposure	N Total population*	n No. of incident strokes	HR	95% CI	p-value	HR	95% CI	p-value
**Active smoking**								**<0.001 **
Never smoker	24,213	193	1	(ref)		1		
Ex/occasional smoker	11,399	172	1.38	1.04 to 1.84	0.026	1.29	0.94 to 1.79	0.119
Current smoker	13,747	220	2.54	1.99 to 3.25	<0.001	2.23	1.67 to 2.98	<0.001
**Alcohol consumption**						Variable dropped**		
Never drinker	2,445	32	1	(ref)				
Ex-drinker	2,927	36	1.03	0.43 to 2.49	0.948			
Light drinker	5,538	83	0.98	0.45 to 2.14	0.950			
Moderate drinker	22,998	228	0.98	0.48 to 1.99	0.957			
Hazardous/harmful drinker	10,365	79	0.96	0.39 to 2.34	0.920			
**Fruit and vegetable consumption**	43,223	403	0.88	0.77 to 1.02	0.088			
**Physical activity**								
High	17,110	96	1			1		
Medium	15,074	141	1.16	0.81 to 1.67	0.400	1.38	0.86 to 2.22	0.179
Low	15,532	313	1.62	1.11 to 2.35	0.013	1.12	0.73 to 1.71	0.605
**Physiological exposures**								
High BMI (per kg/m2)	44,855	527	1.03	1.01 to 1.06	0.017	1.02	1.00 to 1.06	0.054
High total cholesterol (per mmol/l)	20,325	315	0.97	0.88 to 1.07	0.572	Variable dropped**		
High systolic blood pressure (per mm Hg)	23,124	379	1.03	1.01 to 1.05	0.006	1.02	1.01 to 1.03	<0.001

* Total population numbers based on the 20^th^ imputation

** Variable dropped from model as not found to be statistically significant in the age-sex adjusted model.

### Population attributable fractions

Low education accounted for the largest fraction of stroke incidence, explaining 38.8% (95% CI 26.0% to 49.4%) of incident stroke events. This was followed by area deprivation, (as measured by SIMD 2012) which explained 34.9% (95% CI 26.4% to 42.4%) of first stroke and cigarette smoking which explained 25.6% (95% CI 17.9% to 32.6%). High systolic blood pressure explained 29.6% (95%CI 20.6% to 37.6%) while being in an occupational social class other than “professional” explained 30.3% (95%CI 19.4% to 39.8%) of strokes. In addition, a second measure of area deprivation (Carstairs 2001), based on measures of material deprivation, explained 23.5% (95%CI 14.4% to 31.7%) of incident strokes in Scotland.

**Fig 2:**
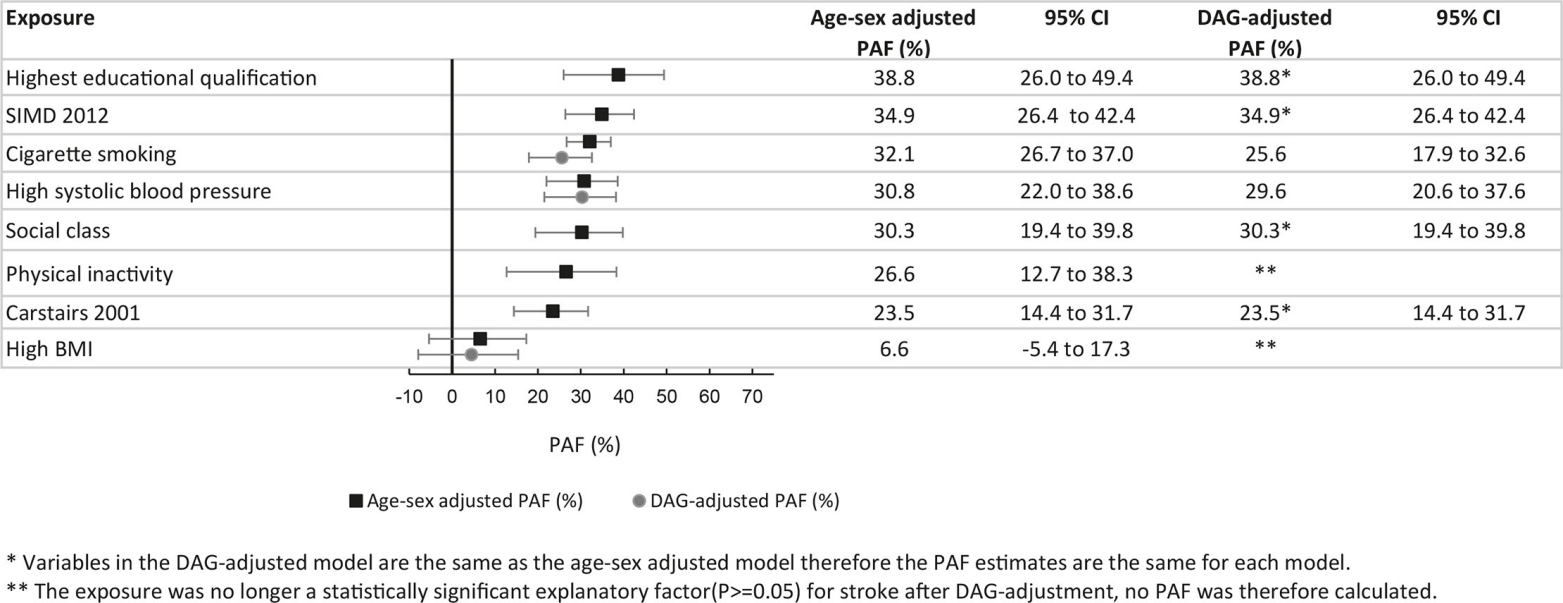
Adjusted PAFs for exposures related to incident stroke—multiple imputation analysis. Based on Cox proportional hazards models.

Fruit and vegetable consumption, BMI and physical activity were significant risk factors for stroke after adjustment for age and sex, but not after full DAG adjustment. Cholesterol, alcohol consumption, unemployment, and equivalised income did not increase the hazard of incident stroke after adjustment for age and sex and were dropped from the model (Fig 2).

### Applying PAFs to Scottish data

PAFs can be applied to actual Scottish incidence data to give the potential reduction in stroke rates that would be expected if the exposure was at the optimum theoretical level. In 2012/13, the age-sex standardised hospital discharge rate for stroke (using the 2013 European Standard Population) was 342.4 per 100,000 population at risk [36] and the number of stroke DALYs was 47,836 [37] (Table 4).

**Table 4:** Potential reduction in age-sex standardised rate of Scottish stroke hospital discharges^1^ ^2^ per 100,000 population and Disability-Adjusted Life-Years (DALY)^3^ in 2012/13 if exposures were reduced to the TMREL.

Exposure	Reduction in crude hospital discharge rate (2012/13)^4^ ^5^	95% CI Discharge rate	Reduction in number of stroke DALY (2013)^6^	95% CI—DALY
Education	132.9	89.0 to 169.0	18,560	12,437 to 23,631
SIMD 2012	119.5	90.4 to 145.2	16,695	12,629 to 20,282
Systolic blood pressure	107.2	72.2 to 137.3	14,973	10,094 to 19,182
Social class	103.7	66.4 to 136.3	14,494	9,280 to 19,039
Smoking	87.7	61.3 to 111.6	12,246	8,562 to 15,595
Carstairs 2001	79.6	48.8 to 107.4	11,241	6,888 to 15,164

^1^ Source of Stroke hospital discharge data: Table AS1: http://www.isdscotland.org/Health-Topics/Stroke/Topic-Areas/Hospital-Activity/

^2^ Hospital discharge data based on all types of admission as of the year ending 31^st^ March

^3^ Source of stroke Disability Adjusted Life Year data: Estimates calculated by and provided by the *Scottish Burden of Disease Project Group*

^4^ Reduction in hospital discharge rate based on a crude discharge rate for all adults of 342.4 discharges per 100,000 population multiplied by respective PAF

^5^ Reduction in the number of DALYs based on a stroke DALY estimate of 47,836 for 2013 (calendar year)

^6^ DAG adjusted PAF estimates used in the calculation of the reduction in stroke crude hospital discharge rate and DALYs.

## Discussion

### Main results

Overall, these results suggest that socio-economic inequality is the strongest explanatory factor for the burden of stroke in Scotland relative to the contribution of the other exposures considered in this study. Of the socio-economic indicators measured, low education explained the largest proportion of incident strokes after DAG adjustment followed by area deprivation (as measured by SIMD), occupational social class and area deprivation (as measured by the Carstairs Index). Of the behavioural and physiological exposures included in this study, cigarette smoking explained the greatest proportion of strokes after DAG-adjustment followed by high systolic blood pressure. Individual PAFs were not summed as they may add to more than 100% due to the possibility of a person being exposed to more than one exposure simultaneously.

In an applied context, this equated to a theoretical reduction in hospital discharge rate from stroke of between 79.6 and 132.9 discharges per 100,000 population at risk (in 2012/2013) through the reduction of socio-economic inequalities and between 87.7 and 107.2 discharges per 100,000 population at risk from a reduction in blood pressure and smoking respectively, if the prevalence of each exposure matched the respective theoretical minimum risk level (TMREL). Similarly, the decrease in the number of stroke Disability Adjusted Life Years (DALYs) which would have theoretically been observed in 2013 from the reduction of socio-economic inequalities to the specified TMREL, would have ranged from 11,241 to 18,560 and between 12,246 and 14,973 from a reduction in the behavioural and physiological exposures described.

### Strengths and limitations

This is the first comparative risk assessment study for stroke in Scotland which includes estimates derived from observed incident cases and which also includes a wide range of exposures across the whole causal pathway. It linked data from a national survey to comprehensive routine datasets (Scottish Morbidity Record 01 (SMR01) and National Records of Scotland (NRS) deaths data) to create a large cohort study. This is a particular strength as it makes causal claims easier as exposure precedes the outcome. It also allows us to directly estimate exposures rather than having to rely on synthetic data.

The Scottish Health Survey (SHeS) provides robust information on a range of behaviours and physiological exposures associated with health outcomes as well as measures of the wider socioeconomic context. The inclusion of socioeconomic exposures into DAGs, and into the subsequent calculation of PAFs, is novel and provides a more comprehensive picture of the range of exposures important in causing stroke.

Two data sources on stroke morbidity were considered for linkage to the Scottish Health Survey. The first of these was the Scottish Stroke Care Audit (SSCA) [6], which collects data on the number of patients admitted to a stroke care unit, those receiving treatment and also outpatient hospital visits from 2002 onwards; the second was the Scottish Morbidity Record (SMR01) [20], with hospital admissions available for stroke going back to 1981. The number of recorded acute strokes in the SSCA and SMR01 varied with SSCA estimates being higher for some hospitals in comparison to SMR01 data and lower in others. Turner [38] found that SMR01 identified 75.2% of stroke incidence in Scotland in 2010–11, in comparison to the SSCA which identified 99.2%. Notwithstanding, SMR01 data were used for this study as this matched more closely time-wise with the Scottish Health Survey; the source of risk factor prevalence data.

This study has a transparent and replicable methodology including the use of Directed Acyclic Graphs (DAGs) to illustrate the causal assumptions underpinning our analyses. The inclusion of 95% confidence intervals around the PAF and DALY estimates added an important measure of precision. This study took the competing risk of death from non-stroke related causes into account by calculating cause-specific hazard rates. This is an important strength of this study as ignoring competing risks can over-estimate the hazard ratios because individuals who die are interpreted statistically as censored i.e. still being able to go on to experience the event of interest.

There is the potential for responder (sample frame and response rate) and recording biases (problems in measuring income, employment, self-assessed alcohol, physical activity and diet). This could lead to a particular bias for exposures such as alcohol which suffer from both responder bias (with heavier drinkers under-represented) and reporting bias (with individuals’ under-reporting how much they actually drink) [39].

There are limitations in the utility of using area deprivation measures for identifying risk at the individual level as not all people in deprived areas will be deprived and not all those in the least deprived areas will be affluent (the ecological fallacy) [40]. The Carstairs Index may not be the best measure of area deprivation as, for example, only 3% of the population are reported to be living in overcrowded conditions and a car is perhaps less of a luxury than in the past, particularly in rural areas where it usually seen as a necessity [41].Both of these issues would tend to bias estimates of the association between socioeconomic position and health outcomes downwards. SIMD has been criticised as an explanatory measure as health is included as part of the index, creating a circularity between exposure and outcome and potentially biasing associations between socioeconomic status and health outcomes upwards. A suggested alternative is to use an index based on the income and employment domains of SIMD. It was not possible to obtain these for the present study, however, it has been shown that the correlation between the income-employment domain and the combined SIMD index is high therefore it is likely that the effect of including the health domain is minimal [40].

The effect of socio-economic deprivation may be mediated through behavioural and physiological exposures such as smoking, high cholesterol and high BMI. The present study did not look at this however due to time constraints.

As discussed in the methods section, Turner et al [38] concluded that stroke incidence in Scotland may be undercounted by up to 25% when estimated using SMR01 (assessed on data for 2010/11). Strokes which do not result in hospitalisation will not be counted in the SMR data, and this may have led to an underestimate of incidence, particularly for earlier data where strokes were more frequently managed in the community. The ICD10 codes used in this study have, however, been shown to have a high Positive Predictive Value (PPV) in epidemiological studies for identifying stroke cases [42]. In addition, strokes that are not hospitalised are potentially less likely to incur as much health loss as those hospitalised due to stroke. As we followed patients up over time, any subsequent strokes resulting in hospitalisation would be captured in the study.

Decisions also had to be made on the direction of causality between exposures, e.g. between alcohol consumption and deprivation. Another salient example is the inclusion of those on medication for health issues such as high blood pressure and cholesterol. Being on medication may artificially lower risk for some people whereas being diagnosed with high blood pressure may motivate an individual to lower their behavioural exposures.

The small number of incident stroke hospitalisations and deaths (n = 585) recorded in the SHeS-SMR-NRS linked dataset may have meant that some analyses were insufficiently powered to detect real differences for some of the exposures. In addition to this, the unbalanced distribution of individuals in different risk categories may account for the inability to detect differences between group categories as described for several exposures earlier.

The risk profile may vary between stroke subtypes due to differing causal pathways. It was not possible to look at this in the current study due to sample size restrictions.

### Comparison with the published literature

The age-specific incidence rate for stroke was approximately four times higher in adults aged 65 years and over, compared to younger adults. This ratio is smaller than that reported in routine administrative data on hospital discharge rates for stroke in 2012/13 with the rate in adults aged 65 years and over approximately 12 times higher than younger adults, a considerably wider gap than suggested by the SHeS-SMR/NRS linked dataset. The exclusion from the SHeS of individuals in institutionalised care, as well as the ‘healthy respondent effect’, whereby those who are not well enough to respond are not therefore represented, may explain why the SHeS sample is generally healthier than the Scottish population as a whole [43].

Five exposure measures were included in the current study in an attempt to capture different elements of socio-economic position (SEP): equivalised household income, SIMD, Carstairs Index, social class and education. The results for each of these will be discussed separately below.

Equivalised household income was dropped from the model as the hazard ratio did not reach significance at the 5% level for any quintiles when compared to the top income quintile (referent). The results from the current study are consistent with two European studies which did not find a link between household income and stroke [44,45]. It is interesting to see the continuation of this patterning of results by the type of income measure as highlighted earlier in the literature review, however, there is a high degree of uncertainty in terms of the strength of evidence presented in these two studies. These two studies both focused on demographically homogenous communities in France and the Netherlands reducing the generalisability to each country as a whole.

The current study used self-reported income data from the SHeS to estimate the income distribution of the Scottish population living in private households. This is a self-reported measure and is known to be problematic as individuals may not wish to divulge their income. A linear increase was observed in hazard ratios with decreasing income and while the effect of income was significant for the model overall (p<0.001), a small, or unequal, sample size (particularly for those in the lowest income quintile) may have resulted in a lack of power to detect the exposure-outcome relationship.

These findings are in contrast to two studies which found an increased risk of stroke in relation to taxable income. The first study [46] had a cohort study design with a very large, registry based sample and 12 years of follow-up. As the study population was restricted to working ages, the generalisability of the results to the wider population may be limited. Jakovlijevic [47] reported a relationship between income and ischaemic stroke based on a population registry covering three regions of Finland. It is difficult to say whether this is representative of the population as a whole. Neither study examined the impact of income on haemorrhagic stroke.

The finding that individuals who reported having no qualifications were more than twice as likely to experience a stroke as those who reported being educated to tertiary level was broadly consistent with the published literature reviewed. Avendano [48] reported a link between low education and increased risk of stroke amongst adults living in England and Wales, however, this association was not evident in adults aged 75 years or older. This might reflect an increased risk of recall bias as individuals get older and the time since leaving education increases.

Of the two measures of area-based deprivation, the percentage of strokes which could be explained by SIMD was higher than that explained by Carstairs (34.9% and 23.5% respectively). This may relate to the different time periods to which these two indices refer (2012 and 2001), a reflection of the data available at the time of analysis, or may represent a difference in the way area deprivation is measured by these two indices. The strength of evidence in the published literature for area deprivation in Scotland is high, particularly given that data on stroke incidence, stratified by SIMD is reported routinely. This provides a good benchmark to compare the SHeS–linked data against.

Occupational social class explained over 30% of stroke events, after adjustment for age and sex. There is some potential for reverse causality as poor health may limit occupational opportunities [49]. It could be argued that social class is not a modifiable exposure, however, the relative power of different social classes, and the benefits and negative consequences of being a part of different social classes, does change profoundly over time and is therefore an important and modifiable series of social processes [50].

Unemployment was not a significant risk factor for incident stroke events in this dataset after adjustment for age and sex though the measure was limited as it did not exclude students or those over retirement age. The question, though consistent across survey waves, does not tell us anything about why a person has not worked in the previous four weeks. This may be due to unemployment but may also be to do with ill health, being a student, or being on some other form of leave from work. A more nuanced measure of unemployment is necessary to tease these issues out more fully.

Four measures of behavioural risk were included in this study (active smoking, alcohol consumption, fruit and vegetable consumption and physical inactivity). There is mixed evidence for the importance of alcohol as an exposure for stroke. Sacco [51] found that the relationship with ischaemic and haemorrhagic stroke varied. The INTERSTROKE study also reported a differential relationship between alcohol consumption and incidence of ischaemic (J-shaped) and haemorrhagic stroke (linear increase) [32]. This highlights the complex relationship between alcohol consumption and stroke risk.

The distribution of drinking habits in the combined SHeS sample was highly variable, with 17,541 individuals reporting moderate drinking patterns compared to 1,966 individuals reporting being never drinkers. The extreme imbalance in numbers between categories may have made it difficult for any differences to be detected between categories despite the overall model effect still being significant at p<0.001. It is also possible, being a self-reported exposure that some reporting bias exists in this survey. It is also widely reported that the health surveys underestimate alcohol consumption [39].

Active smoking was the only behavioural exposure which was still associated with incident stroke events following adjustment for potential confounders. The 1995 and 1998 SHeS waves contributed more than 50% of the person-years at risk. The results may not therefore account for the impact of Scottish smoke-free legislation on smoking levels.

Smoking has consistently been reported as an important predictor of increased stroke risk in the published literature [52–54]. Fahimfar [54] reported PAFs for the proportion of strokes attributable to smoking at results lower than ours between 12 and 14% compared to 25.6% in the present study. The highest PAF found in the published literature was by Scarborough [55] at 22%, which is consistent with our estimate. These differences may be partially explained by varying prevalence rates and measures of smoking used in these studies.

Though the present study did not find physical activity to be a significant predictor of stroke after full adjustment for confounding, there is published evidence to support a protective effect of physical activity on stroke incidence [52]. The measure used in the current study was self-reported which may have introduced reporting bias.

In contrast to the results in this study which found no causal link between stroke and fruit and vegetable consumption, the World Health Organization estimated that low fruit & vegetable consumption explained 11% of strokes worldwide [56].

A PAF was not calculated for BMI based on the DAG-adjusted model as this showed only borderline statistical significance (p = 0.054). Other studies have reported a significant causal link between high BMI and stroke which is not highly inconsistent with our findings [57, 58].

Of the three physiological exposures measured in this study, high systolic blood pressure was most strongly linked to stroke incidence. This is consistent with several previous studies [32,52,56,57]. The measure of systolic blood pressure did not exclude those on blood pressure reducing drugs, so there is the potential for misclassification of risk as those with blood pressure at normal levels due to medication may have already suffered physiological damage through previous exposure, masking their true risk profile. A sensitivity analysis excluding those reporting being on treatment would be a useful measure.

Total cholesterol was not a significant predictor of stroke in our study. The relationship between cholesterol and stroke in the published literature was mixed. Sacco [51] reported the link to ischaemic stroke as being inconclusive [52]. The INTERSTROKE study [32] reported that increased concentration of HDL cholesterol was associated with a reduced risk of ischaemic stroke but an increased risk of haemorrhagic stroke. This also highlights the importance of considering exposures for stroke subtypes where possible.

### Conclusions

The results from this comparative risk assessment approach provide evidence for the relative importance of a range of modifiable exposures in explaining stroke. They provide evidence to suggest that the greatest public health gains from reducing morbidity and mortality from stroke are to be made from prioritising interventions that target socio-economic deprivation. Physiological and behavioural exposures such as high systolic blood pressure and smoking were also important explanatory factors for stroke and offer additional opportunities to reduce the burden of this disease in Scotland. The methodological approach used in this study also lays the foundation for future work to explore the role of mediating pathways between exposure and stroke. This may be important from a policy perspective if we can strengthen our understanding of the relative importance of intermediate variables as mediators along the causal pathway. In addition, there is a need to better understand the type of interventions that would be most effective in realising the potential gains estimated through comparative risk assessment studies.

## Supporting information

S1 Fig Directed Acyclic Graph for exposure to deprivation (SIMD). (EPS)

S2 Fig Directed Acyclic Graph for exposure to smoking. (EPS)

S3 Fig Directed Acyclic Graph for exposure to alcohol consumption. (EPS)

S4 Fig Directed Acyclic Graph for exposure to low fruit/vegetable consumption. (EPS)

S5 Fig Directed Acyclic Graph for exposure to physical inactivity. (EPS)

S6 Fig Directed Acyclic Graph for exposure to high body mass index (BMI). (EPS)

S7 Fig Directed Acyclic Graph for exposure to high systolic blood pressure. (EPS)

S8 Fig Directed Acyclic Graph for exposure to high cholesterol. (EPS)

S1 File Web supplement. Additional methodological details.
(DOCX)

## References

[1] Grant I, Wyper G. The use of routine health data and record linkage in a National Burden of Disease Study for Scotland. In: FARR Conference 2015. St Andrew's, Farr Institute and University of St Andrew's, 2015.

[2] Vos T, Barber RM, Bell B, Bertozzi-Villa A, Biryukov S, Bolliger I, et al Global, regional, and national incidence, prevalence, and years lived with disability for 301 acute and chronic diseases and injuries in 188 countries, 1990–2013: a systematic analysis for the Global Burden of Disease Study 2013. The Lancet 2015 10.1016/S0140-6736(15)60692-4 26063472 PMC4561509

[3] Lopez A, Mathers CE, Ezzati M, Jamison D, Murray C (editors). Global Burden of Disease and Risk Factors New York, Oxford University Press, 2006.

[4] Forouzanfar MH, Afshin A, Alexander LT, Anderson HR, Bhutta ZA, Biryukov S, et al Global, regional, and national comparative risk assessment of 79 behavioural, environmental and occupational, and metabolic risks or clusters of risks, 1990–2015: a systematic analysis for the Global Burden of Disease Study 2015. The Lancet 2017;388(10053):1659–1724. 10.1016/S0140-6736(16)31679-8 PMC5388856 27733284

[5] Forouzanfar MH, Moran A, Phillips D, Mensah G, Ezzati M, Naghavi M, et al Prevalence of heart failure by cause in 21 regions: Global burden of diseases, injuries and risk factors-2010 study. Journal of the American College of Cardiology. Conference: 62nd Annual Scientific Session of the American College of Cardiology and i2 Summit: Innovation in Intervention, ACC.13 San Francisco, CA United States,2013;61(10 SUPPL. 1):E786.

[6] Public Health Intelligence, National Health Service. Scottish Stroke Care Audit. 2015; Available at: http://www.strokeaudit.scot.nhs.uk/about.htm. Accessed 10/01, 2015.

[7] Di Carlo A. Human and economic burden of stroke. Age and ageing 2009;38:4. 10.1093/ageing/afn282 19141505

[8] Institute for Health Metrics and Evaluation. Country profiles: United Kingdom—Scotland. 2017; Available at: http://www.healthdata.org/united-kingdom-scotland. Accessed May/01, 2018.

[9] Scottish Burden of Disease Team. Scottish Burden of Disease Data 2015. 2017; Available at: http://www.scotpho.org.uk/comparative-health/burden-of-disease/sbod-data-2015. Accessed 11/09, 2017.

[10] Lewsey J, Jhund P, Gillies M, Chalmers J, Redpath A, Kelso L, et al Age and sex specific trends in fatal incidence and hospitalised incidence of stroke in Scotland, 1986 to 2005. Circulation, Cardiovascular Quality & Outcomes 2009;2(5):475. 20031880 10.1161/CIRCOUTCOMES.108.825968

[11] Information Services Division. Cerebrovascular Disease: Trends in mortality 2003–2012. 2014; Available at: http://www.isdscotland.org/Health-Topics/Stroke/Topic-Areas/Mortality/. Accessed 09/02, 2014.

[12] Whyte B, Ajetunmobi T. Still "The sick man of Europe"? Scottish Mortality in a European Context. 1950–2010 An analysis of comparative mortality trends. Glasgow, Glasgow Centre for Population Health, 2012.

[13] Information Services Division, NHS Scotland. Stroke: Survival (January 2014 release). Edinburgh, NHS National Services Scotland, 2014.

[14] NHS Choices. Stroke. 2014; Available at: http://www.nhs.uk/Conditions/Stroke/Pages/Introduction.aspx. Accessed 09/02, 2014.

[15] Bhatnagar P, Scarborough P, Smeeton N, Allender S. The incidence of all stroke and stroke subtype in the United Kingdom, 1985 to 2008: a systematic review. BMC Public Health 2010;10(1):539. 20825664 10.1186/1471-2458-10-539 PMC2944372

[16] Malmivaara A, Meretoja A, Peltola M, Numerato D, Heijink R, Engelfriet P, et al Comparing ischaemic stroke in six European countries. The EuroHOPE register study. European Journal of Neurology 2015; 22(2):284–91, e25-6. 10.1111/ene.12560 25196190

[17] Brown L, Campbell-Jack D, Gray L, Hovald P, Kirkpatrick G, Knudsen L, et al. The Scottish Health Survey 2015: Volume 1: Main Report. Edinburgh, Scottish Government, 2016. Available at: http://www.gov.scot/Publications/2016/09/2764. Accessed May/01, 2018.

[18] StataCorp. Stata Statistical Software: Release 13. College Station, TX: StataCorp LP. 2013.

[19] Administrative Data Liaison Service. Scottish Health Survey. Edinburgh, Scottish Government, 2015. Available at: http://www.adls.ac.uk/find-administrative-data/linked-administrative-data/scottish-health-survey/.

[20] ISD Scotland. SMR01—General / Acute Inpatient and Day Case. Edinburgh, NHS National Services Scotland, 2017. Available at: http://www.ndc.scot.nhs.uk/Data-Dictionary/SMR-Datasets/SMR01-General-Acute-Inpatient-and-Day-Case/.

[21] Whitehead M, Dahlgren G. Concepts and principles for tackling social inequities in health: Levelling up Part 1. Copenhagen: World Health Organisation; 1991.

[22] UNESCO: Institute of Statistics. ISCED mappings (1997): ISCMAP-QUAL framework (UK). Montreal, UNESCO, 2014. Available at: http://www.uis.unesco.org/Education/ISCEDMappings/Pages/default.aspx. Accessed 10/01, 2015.

[23] SIMD Scottish Index of Multiple Deprivation. Edinburgh, Scottish Government, 2012. Available at: http://simd.scotland.gov.uk/publication-2012/simd-2012-results/domain-results/employment-domain/. Accessed 07/01, 2014.

[24] STATA.com. mi impute chained—Impute missing values using chained equations. College Station (Texas), STATA, 2013. Available at: http://www.stata.com/manuals13/mimiimputechained.pdf. Accessed 08/24, 2015.

[25] Rodriguez G. Parametric Survival Models. Princeton, Princeton University, 2010. Available at: http://data.princeton.edu/pop509/ParametricSurvival.pdf. Accessed: 9th November 2017.

[26] Shrier I, Platt RW. Reducing bias through directed acyclic graphs. BMC Med Res Methodol 2008;8:7 10.1186/1471-2288-8-7 18973665 PMC2601045

[27] Textor J, Hardt J, Knuppel S. DAGitty: a graphical tool for analyzing causal diagrams. Epidemiology 2011;22(5):745. 10.1097/EDE.0b013e318225c2be 21811114

[28] Lim SS, Vos T, Flaxman AD, Danaei G, Shibuya K, Adair-Rohani H, et al A comparative risk assessment of burden of disease and injury attributable to 67 risk factors and risk factor clusters in 21 regions, 1990–2010: a systematic analysis for the Global Burden of Disease Study 2010. The Lancet 2017;380(9859):2224–2260. 10.1016/S0140-6736(12)61766-8 PMC4156511 23245609

[29] GBD 2013 Mortality and Causes of Death Collaborators. Global, regional, and national age-sex specific all-cause and cause-specific mortality for 240 causes of death, 1990–2013: A systematic analysis for the Global Burden of Disease Study 2013. The Lancet 2014 10 1 2015;385(9963):117–171. 10.1016/S0140-6736(14)61682-2 PMC4340604 25530442

[30] Feigin VL, Roth GA, Naghavi M, Parmar P, Krishnamurthi R, et al Global burden of stroke and risk factors in 188 countries, during 1990–2013: a systematic analysis for the Global Burden of Disease Study 2013. Lancet Neurology 2016; 15(9): 913–924. 10.1016/S1474-4422(16)30073-4 27291521

[31] Feigin VL, Forouzanfar MH, Krishnamurthi R, Mensah GA, Connor M, Bennett DA, et al Global and regional burden of stroke during 1990–2010: findings from the Global Burden of Disease Study 2010. The Lancet 2014;383(9913):245–255. 10.1016/s0140-6736(13)61953-4 PMC4181600 24449944

[32] O'Donnell MJ, Xavier D, Liu L, Zhang H, Chin SL, Rao-Melacini P, et al Risk factors for ischaemic and intracerebral haemorrhagic stroke in 22 countries (the INTERSTROKE study): a case-control study. The Lancet 2010;376(9735):112–123. 10.1016/S0140-6736(10)60834-3 20561675

[33] Greenland S, Drescher K. Maximum likelihood estimation of the attributable fraction from logistic models. Biometrics 1993;49(3):865–872. 8241375

[34] Newson R. Attributable and unattributable risks and fractions and other scenario comparisons. The STATA Journal 2013;13(4):672.

[35] ScotPHO. National Burden of Disease, Injuries and Risk Factors Study. Edinburgh, ScotPHO, 2015. Available at: http://www.scotpho.org.uk/comparative-health/burden-of-disease. Accessed 10/05, 2015.

[36] Information Services Division. Stroke Statistics Update Year Ending 31 March 2014. Edinburgh, NHS National Services Scotland, 2015. Available at: https://isdscotland.scot.nhs.uk/Health-Topics/Stroke/Publications/2015-01-27/2015-01-27-Stroke-Report.pdf?76363772154. Accessed 10/01, 2015.

[37] Grant I, McFadden M, Wyper G, Stockton D, McCartney G, Fischbacher C, et al Scottish Burden of Disease Study. Stroke Disability Adjusted Life Years: methods and results. Edinburgh, Scottish Burden of Disease Study Project Team 2015.

[38] Turner M, MacLeod M. Using SSCA data for research. Edinburgh, NHS National Services Scotland, 2014. Available at: http://www.strokeaudit.scot.nhs.uk/Meetings/Presentations-2014/_docs/Using-SSCA-data-for-research.pdf. Accessed 10/02, 2015.

[39] Gray L, McCartney G, White IR, Katikireddi SV, Rutherford L, Gorman E, et al Use of record-linkage to handle non-response and improve alcohol consumption estimates in health survey data: a study protocol. BMJ Open 2013;3:e002647 10.1136/bmjopen-2013-002647 23457333 PMC3612815

[40] Fischbacher C. Identifying “deprived individuals”: are there better alternatives to the Scottish Index of Multiple Deprivation (SIMD) for socioeconomic targeting in individually based programmes addressing health inequalities in Scotland? Edinburgh, NHS National Services Scotland, 2014. Available at: http://www.scotpho.org.uk/downloads/scotphoreports/scotpho140109-simd-identifyingdeprivedindividuals.pdf. Accessed 01/10, 2015.

[41] Brown D, Allik M, Dundas R, Leyland A. Carstairs Scores for Scottish Postcode Sectors, Datazones & Output Areas from the 2011 Census. Glasgow, CSO/MRC Social and Public Health Sciences Unit, 2014. Available at: f www.sphsu.mrc.ac.uk/carstairs_2011_report.pdf. Accessed 10/30, 2015.

[42] Woodfield R, Grant I, UK Biobank Stroke OG, UK Biobank Follow-Up and Outcomes Working Group, Sudlow CLM. Accuracy of Electronic Health Record Data for Identifying Stroke Cases in Large-Scale Epidemiological Studies: A Systematic Review from the UK Biobank Stroke Outcomes Group. PLOS ONE 2015;10(10):e0140533 10.1371/journal.pone.0140533 26496350 PMC4619732

[43] McCartney G, Russ TC, Walsh D, Lewsey J, Smith M, Davey Smith G, et al Explaining the excess mortality in Scotland compared with England: pooling of 18 cohort studies. Journal of Epidemiology and Community Health 2015;69:20–27. 10.1136/jech-2014-204185 25216666 PMC4283682

[44] Grimaud O, Béjot Y, Heritage Z, Vallée J, Durier J, Cadot E, et al Incidence of Stroke and Socioeconomic Neighborhood Characteristics: An Ecological Analysis of Dijon Stroke Registry. Stroke 2011;42(5):1201–1206. 10.1161/STROKEAHA.110.596429 21393599

[45] van Rossum CTM, van de Mheen H, Breteler MMB, Grobbee DE, Mackenbach JP. Socioeconomic Differences in Stroke Among Dutch Elderly Women: The Rotterdam Study. Stroke 1999;30(2):357–362. 9933271 10.1161/01.str.30.2.357

[46] Toivanen S. Income differences in stroke mortality: A 12-year follow-up study of the Swedish working population. Scandinavian Journal of Public Health 2011;39(8):797–804. 10.1177/1403494811418280 21893604

[47] Jakovljevic D, Sarti C, Sivenius J, Torppa J, Mähönen M, Immonen-Räihä P, et al Socioeconomic Status and Ischemic Stroke: The FINMONICA Stroke Register. Stroke 2001;32(7):1492–1498. 11441191 10.1161/01.str.32.7.1492

[48] Avendano M, FAU KA, van Lenthe FF, Bos VF, Costa GF, Valkonen TF, et al Trends in socioeconomic disparities in stroke mortality in six european countries between 1981–1985 and 1991–1995. American journal of epidemiology 2005;161(1):52–61. 10.1093/aje/kwi011 15615915

[49] Cox AM, McKevitt C, Rudd AG, Wolfe CD. Socioeconomic status and stroke. The Lancet Neurology 2006 2;5(2):181–188. 10.1016/S1474-4422(06)70351-9 16426994

[50] Wright E O. Envisioning Real Utopias. London: Verso; 2010.

[51] Sacco RL, Kasner SE, Broderick JP, Caplan LR, Connors JJ(, Culebras A, et al An Updated Definition of Stroke for the 21st Century: A Statement for Healthcare Professionals From the American Heart Association/American Stroke Association. Stroke 2013;44(7):2064–2089. 10.1161/STR.0b013e318296aeca 23652265 PMC11078537

[52] Panel Sacco RL, Benjamin EJ Broderick JP, Dyken M Easton JD, et al Risk Factors. Stroke 1997;28(7):1507–1517. 9227708 10.1161/01.str.28.7.1507

[53] Murray C, Ezzati M, Lopes A, Rodgers A, Vander Hoorn S. Comparative quantification of health risks: Conceptual framework and methodological issues. Population Health Metrics 2003(1):1 10.1186/1478-7954-1-1 12780936 PMC156894

[54] Fahimfar N, Khalili DF, Mohebi RF, Azizi FF, Hadaegh F. Risk factors for ischemic stroke; results from 9 years of follow-up in a population based cohort of Iran. BMC neurology 2012; 12:117 10.1186/1471-2377-12-117 23031547 PMC3517457

[55] Scarborough P, Bhatnagar P, Wickramasinghe K, Allender S, Foster C, Rayner M. The economic burden of ill health due to diet, physical inactivity, smoking, alcohol and obesity in the UK: an update to 2006–07 NHS costs. Journal of Public Health 2011; 33(4):527–35. 10.1093/pubmed/fdr033 21562029

[56] World Health Organization. Risk Factors. Geneva, WHO, 2003 Available at: http://www.who.int/cardiovascular_diseases/en/cvd_atlas_03_risk_factors.pdf, 201510/01.

[57] Bos MJ, Koudstaal PJ, Hofman A, Ikram MA. Modifiable Etiological Factors and the Burden of Stroke from the Rotterdam Study: A Population-Based Cohort Study. PLoS Med 2014;11(4):e1001634 10.1371/journal.pmed.1001634 24781247 PMC4004543

[58] Mitchell AB, Cole JW, McArdle PF, Cheng Y, Ryan KA, Sparks MJ, et al Obesity Increases Risk of Ischemic Stroke in Young Adults. Stroke 2015;46(6):1690–1692. 10.1161/STROKEAHA.115.008940 25944320 PMC4458137

