# Supplementary material for: What causes the burden of stroke in Scotland? A comparative risk assessment approach linking the Scottish Health Survey to administrative health data
Source: PLoS One. 2019 Jul 8;14(7):e0216350. doi: 10.1371/journal.pone.0216350 (PMC6613691; doi:10.1371/journal.pone.0216350)
Supplement: S1 File — Additional methodological details. (DOCX) [file pone.0216350.s009.docx]

**S1 File – Additional methodological details**

**Exposure selection**

| **Dahlgren & Whitehead layer** | **Possible exposures** | **Availability of exposure data** | **Importance** | **Feasibility of modification** |
| --- | --- | --- | --- | --- |
| General socioeconomic, cultural and environmental conditions | Climate change | Not available | High | Low |
|  | Air pollution | Not available | High | Moderate |
|  | Water pollution | Not available | Low | Moderate |
|  | Land contamination | Not available | Low | Moderate |
|  | Low income | Linked SHeS | High | Low |
|  | Wealth | Not linked | High | Low |
|  | Area deprivation | Linked SHeS | High | Low |
| Living & working conditions | Low education | Linked SHeS | High | Moderate |
|  | Unemployment | Linked SHeS | High | Moderate |
|  | Poor housing | Not available | High | Moderate |
| Social & community networks | Social isolation/social capital | Not available | High | Unclear |
| Individual lifestyle factors | Tobacco smoking | Linked SHeS | High | High |
|  | Alcohol consumption | Linked SHeS | High | Moderate |
|  | No/low fruit and vegetables | Linked SHeS | High | Moderate |
|  | Illicit drugs | Not available | High | Moderate |
|  | Physical inactivity & low physical activity | Linked SHeS | High | Unclear |
| Physiological factors | High total cholesterol | Linked SHeS | High | High |
|  | High body-mass index | Linked SHeS | High | Low |
|  | High systolic blood pressure | Linked SHeS | High | High |

| **Classification** | **Criteria** |
| --- | --- |
| No | Not amenable to change e.g. age |
| Low | Potentially amenable to change in the longer term through changes in culture, policy or practice. |
| Moderate | Potentially amenable to change in the mid-term through changes to policy, practice or lifestyle changes. |
| High | Potentially amenable to change in the short-medium term through changes to policy, practice or lifestyle changes. |

**Minimally Sufficient Adjustment Set (MSAS) for total effect of each selected risk factor on first stroke incidence i.e. DAG model**

| **Exposure** | **MSAS for estimating the total effect of**  **each respective exposure on stroke incidence** |
| --- | --- |
| SIMD 2012 | None |
| Carstairs 2001 | None |
| Low equivalised income | None |
| Unemployment | None |
| Low education | None |
| Smoking | Carstairs 2001  SIMD 2012  low education  low income  unemployment |
| Alcohol consumption | Carstairs 2001  SIMD 2012  Low education  Low income  Unemployment |
| No/low fruit and vegetables | Carstairs 2001  SIMD 2012  Low education  Low income  Unemployment |
| High cholesterol | Carstairs 2001  SIMD 2012  High BMI  Low education  Low income  Unemployment |
| High BMI | Carstairs 2001  SIMD 2012  High total cholesterol  Low education  Low income  Unemployment |
| High systolic blood pressure | High BMI  SIMD 2012  Low education  Low income  Unemployment  Smoking |

**Number of cases with ‘soft’ and ‘hard’ missing data for each risk factor variable**

| Variable | Survey waves with valid measure | Non-missing (all waves 1995-2012) | No. soft missing* (all waves 1995-2012) | % of observations soft missing  (all waves  1995-2012) | Multiple imputation method |
| --- | --- | --- | --- | --- | --- |
| Equivalised income | 2003-2012 | 30,584 | 15,096 | 30.6 | Ordered logistic regression |
| SIMD 2012 | 1995-2012 | 49,411 | 40 | 0.1 | Ordered logistic regression |
| Carstairs 2001 | 1995-2012 | 49,411 | 40 | 0.1 | Ordered logistic regression |
| Social class | 1995-2012 | 46,928 | 166 | 0.3 | Ordered logistic regression |
| Education | 1995-2012 | 47,198 | 2,253 | 4.3 | Ordered logistic regression |
| Unemployment | 1995-2012 | 42,361 | 7,090 | 14.4 | Logistic regression |
| Smoking | 1995-2012 | 49,351 | 93 | 0.2 | Ordered logistic regression |
| Alcohol consumption | 2003-2012** | 34,069 | 15,373 | 31.1 | Ordered logistic regression |
| Fruit & vegetables | 2003-2012 | 34,356 | 15,087 | 30.5 | Linear regression |
| BMI | 1995-2012 | 43,598 | 5,345 | 10.8 | Linear regression |
| Physical activity | 1998-2012 | 42,315 | 7,136 | 14.4 | Ordered logistic regression |
| Cholesterol | 1995-2011 | 17,106 | 5,832 | 11.8 | Linear regression |
| Systolic blood pressure | 1995-2011 | 18,937 | 5,791 | 11.7 | Linear regression |

* Missing means those who were eligible for inclusion but did not through non-response and excludes those for whom the question was not applicable

** Measure introduced in 2003 along with revision of alcohol strengths. Values for 1995 and 1998 to be imputed for comparability

**Number of complete, incomplete and impute observations per imputation (m)**

|  | **Observations per *m*** | | |  |
| --- | --- | --- | --- | --- |
| **Risk factor** | **Complete** | **Incomplete** | **Imputed** | **Total** |
| **BMI** | 43,598 | 5,345 | 1,257 | 48,943 |
| **Total cholesterol** | 17,106 | 5,832 | 3,219 | 22,938 |
| **Systolic blood pressure** | 18,937 | 5,791 | 4,187 | 24,728 |
| **Fruit & vegetables** | 34,364 | 15,087 | 10,168 | 49,451 |
| **Equivalised income** | 30,584 | 15,096 | 10,169 | 45,680 |
| **Cigarette smoking** | 49,351 | 93 | 8 | 49,444 |
| **Alcohol consumption** | 34,069 | 15,373 | 10,204 | 49,442 |
| **SIMD 2012** | 49,411 | 40 | 15 | 49,451 |
| **Carstairs 2001** | 49,411 | 40 | 15 | 49,451 |
| **Social class** | 46,938 | 166 | 97 | 47,104 |
| **Highest qualification** | 47,198 | 2,253 | 404 | 49,451 |
| **Physical activity** | 42,315 | 7,136 | 5,401 | 49,451 |
| **Unemployment** | 42,361 | 7,090 | 5,391 | 49,451 |

**Proportions at each level for categorical variables (observed, imputed & complete datasets)**

| **Categorical risk factor** | **Proportion in observed dataset** | **Proportion in imputed dataset** | **Proportion in completed dataset** |
| --- | --- | --- | --- |
| **Equivalised income** |  |  |  |
| Top quintile | 0.222 | 0.183 | 0.212 |
| 2^nd^ quintile | 0.213 | 0.197 | 0.209 |
| 3^rd^ quintile | 0.199 | 0.220 | 0.204 |
| 4^th^ quintile | 0.201 | 0.224 | 0.207 |
| 5^th^ quintile | 0.165 | 0.177 | 0.168 |
| **SIMD (2012)** |  |  |  |
| 1^st^ (most deprived quintile) | 0.203 | 0.267 | 0.203 |
| 2^nd^ | 0.203 | 0.067 | 0.203 |
| 3^rd^ | 0.207 | 0.133 | 0.207 |
| 4^th^ | 0.210 | 0.400 | 0.210 |
| 5^th^ (least deprived quintile) | 0.178 | 0.133 | 0.178 |
| **Carstairs (2001)** |  |  |  |
| 1^st^ (least deprived quintile) | 0.188 | 0.133 | 0.188 |
| 2^nd^ | 0.235 | 0.267 | 0.235 |
| 3^rd^ | 0.211 | 0.067 | 0.211 |
| 4^th^ | 0.185 | 0.267 | 0.185 |
| 5^th^ (most deprived quintile) | 0.181 | 0.267 | 0.181 |
| **Social class** |  |  |  |
| I. Professional | 0.049 | 0.062 | 0.049 |
| II. Managerial - Technical | 0.282 | 0.289 | 0.282 |
| IIIN. Skilled – non manual | 0.228 | 0.278 | 0.228 |
| IIIM. Skilled - manual | 0.193 | 0.165 | 0.193 |
| IV. Semi-skilled - manual | 0.180 | 0.144 | 0.180 |
| Unskilled | 0.069 | 0.062 | 0.069 |
| **Education** |  |  |  |
| Tertiary level | 0.228 | 0.453 | 0.299 |
| Post-secondary level | 0.079 | 0.042 | 0.078 |
| Upper-secondary level | 0.150 | 0.099 | 0.150 |
| Lower secondary level | 0.245 | 0.292 | 0.245 |
| No qualifications | 0.298 | 0.453 | 0.299 |
| **Unemployment** |  |  |  |
| No | 0.440 | 0.311 | 0.426 |
| Yes | 0.560 | 0.689 | 0.574 |
| **Smoking** |  |  |  |
| Never | 0.491 | 0.250 | 0.490 |
| Ex/Occasional | 0.231 | 0.125 | 0.231 |
| Current | 0.279 | 0.625 | 0.279 |
| **Alcohol consumption** |  |  |  |
| Never drinker | 0.058 | 0.043 | 0.054 |
| Ex-drinker | 0.067 | 0.052 | 0.064 |
| Light drinker | 0.128 | 0.113 | 0.124 |
| Moderate drinker | 0.515 | 0.530 | 0.518 |
| Hazardous/harmful drinker | 0.232 | 0.261 | 0.239 |
| **Physical activity** |  |  |  |
| Low | 0.330 | 0.275 | 0.324 |
| Medium | 0.314 | 0.328 | 0.316 |
| High | 0.356 | 0.397 | 0.361 |

**Distribution of observed, imputed and complete observations for the four continuously measured risk factors: fruit and vegetable consumption, BMI, systolic blood pressure & cholesterol**
